# Hajj-Related *Neisseria Meningitidis* Serogroup W135 in Mauritius

**DOI:** 10.3201/eid0803.010372

**Published:** 2002-03

**Authors:** Mohammad Iqbal Issack, Chinien Ragavoodoo

**Affiliations:** * Central Health Laboratory, Ministry of Health, Mauritius; † Communicable Disease Unit, Ministry of Health, Mauritius

**Keywords:** Neisseria meningitidis W135, Mauritius, Hajj pilgrims

## Abstract

Meningococcal disease is rare in Mauritius; only one case was reported from 1992 to 1999. However, since June 2000, four cases have occurred. Epidemiologic information and typing results indicate that these recent cases probably followed the introduction of *Neisseria meningitidis* W135 in Mauritius by pilgrims returning from the Hajj in 2000 and 2001.

Mauritius is a small tropical island in the Indian Ocean (population 1.2 million). The country is classified as a middle-income country by the World Bank; its primary commercial links are with Europe, the Indian subcontinent, and southern Africa.

Bacteriologic investigations for government health institutions in Mauritius are conducted in only one laboratory. These centralized results indicate that meningococcal disease is extremely rare in Mauritius. From 1992 to 1999, the only recorded infection occurred in a patient 3 days after he returned from the United Kingdom. However, since June 2000, four cases of meningococcal disease have occurred in Mauritians who have no history of travel outside the island.

## Case Reports

### Case 1

In June 2000, a 49-year-old Muslim woman was admitted to the hospital with a purpuric rash. She was initially thought to have a hematologic disorder before meningococcal septicemia was suspected. Despite treatment with intravenous penicillin and cefotaxime, she died the following day. *Neisseria meningitidis* was isolated from her blood cultures. A latex agglutination test with polyvalent reagent was positive for groups ACYW135 (Wellcogen bacterial antigen kit, Murex, Dartford, UK), but negative results were obtained with monovalent sera for group A, B, and C antigens (Slidex, Biomérieux, Marcy l’Etoile, France). The isolate was subsequently typed by the World Health Organization (WHO) Collaborating Centre for Meningococcal Infections in Marseilles, where it was confirmed as *N. meningitidis* serogroup W135, type 2a, subtype 1.2,5. It belonged to ST-11, the same sequence type as isolates obtained from English and French pilgrims returning from the Hajj that same year (*1*). The patient had no history of travel or close contact with a returning pilgrim, according to relatives.

### Case 2

In July 2000, a 5-year-old Muslim child was admitted to the hospital with fever, vomiting, and ecchymoses. Initially she was thought to have a bleeding disorder. She had already received intravenous amoxicillin when meningococcal disease was suspected and specimens for microbiologic investigation taken. Her cerebrospinal fluid (CSF) was turbid with a leukocyte count of 11,500 per µL. CSF was positive for *N. meningitidis* antigens, groups ACYW135 with polyvalent serum, and negative for *Haemophilus influenzae* type b, pneumococcus, and meningococcus groups A, B, and C. Cultures of CSF and blood were negative. Cefotaxime was added to her treatment, and she made a good recovery.

The child’s father, who returned from the Hajj 3 months earlier, had received the meningococcus A+C bivalent vaccine before travel. He had not been clinically ill during the pilgrimage or after his return.

### Case 3

In November 2000, a 27-year-old man was admitted to the hospital with ecchymoses and signs and symptoms of meningitis. He had received antibiotics before investigations were performed. His CSF was turbid, with 26,000 leukocytes and 10,000 red cells per µL. Results of antigen testing on blood and CSF specimens were similar to those of the child in case 2, and cultures were negative. He was treated with cefotaxime and fully recovered. The patient was not Muslim and had not had close contact with a returning pilgrim. However, he and the patient in case 1 lived in the same village (population 10,000) as 22 pilgrims from the 2000 Hajj.

### Case 4

In April 2001, a 4-month-old Muslim infant was hospitalized with fever and ecchymoses. Her CSF was turbid with 900 leukocytes per µL, and *N. meningitidis* was confirmed by culture. She was treated with cefotaxime and made a full recovery. Her father had returned from the Hajj 2 weeks before her onset of symptoms, but apart from a cough and cold, he had been clinically well. However, meningococcus was isolated from his oropharynx, as well as from the throat of the patient’s 2-year-old brother. Isolates from the patient and her father and brother were positive by agglutination with meningococcus ACYW135 polyvalent reagent and negative with monovalent sera for meningococcus A, B, and C.

 The pilgrim’s vaccination certificate confirmed that he had received a quadrivalent meningococcal vaccine. All three isolates were sent to the WHO Collaborating Centre, which confirmed meningococcus serogroup W135, type 2a, subtype 1.2,5. They were indistinguishable by pulsed-field gel electrophoresis from isolates obtained from French pilgrims in 2000.

Close contacts of all four patients with meningococcal disease received antimicrobial prophylaxis within 24 hours of diagnosis: a single 500-mg dose of ciprofloxacin was given to adults, and 2 days of rifampicin (10 mg/kg twice a day) was given to children. Close contacts of the infant (case 4) were also given the quadrivalent meningococcal vaccine. The only meningococcal carriage studies were conducted on some close contacts of this infant. No case secondary to those reported above is known to have occurred.

Two of these patients had meningococcal disease that was shown by typing to have been caused by the same W135 clone isolated from pilgrims returning from the Hajj in 2000 and 2001. Case 2 was culture negative but epidemiologically linked with a returning pilgrim and was probably caused by the same clone. Case 3 was also culture negative, but antigen tests indicated that infection was caused by meningococcus group W135 or group Y.

## Conclusions

Cases of W135 meningococcal disease in returning pilgrims and their contacts have been reported in several countries following the Hajj of 2000 (*1*) and 2001 (*2*). In many European countries, further cases later occurred in persons with no history of close contact with a returning pilgrim (*3*).

About 16% of the Mauritian population are Muslims, and approximately 1,800 and 2,200 pilgrims traveled from Mauritius to Saudi Arabia for the Hajj in 2000 and 2001, respectively. In 2000, Mauritian pilgrims received the bivalent A+C meningococcal polysaccharide vaccine, but five Mauritians reportedly died in Saudi Arabia of meningitis of unspecified type. Cases of meningococcal disease in Mauritius itself occurred 3 months after the pilgrims returned, a period that coincided with the annual peak in upper respiratory tract infections (*4*). Viral upper respiratory tract infections are known to increase the risk of meningococcal disease (*5*). After the infections were reported in 2000, the Mauritian government decided to import a quadrivalent meningococcal polysaccharide vaccine (Mencevax ACWY, SmithKline Beecham, Genval, Belgium) for pilgrims attending the Hajj in 2001, and almost all of them received that vaccine. Although no case of meningitis was subsequently reported among these pilgrims, case 4 suggests that some have become carriers of meningococcus W135.

Evidence indicates that the quadrivalent meningococcal polysaccharide vaccine may not prevent asymptomatic nasopharyngeal infection with *N. meningitidis* serogroup W135 (*6*). Prophylactic administration of antibiotics to returning pilgrims may be indicated to reduce the risk for transmission to close contacts. A recent study in the United States showed that 0.8% of 727 returning pilgrims in 2001 were carriers of W135 meningococcus, compared with none on departure (*7*). All these pilgrims are presumed to have received the quadrivalent meningococcal vaccine. In view of the low carriage rate, administration of chemoprophylaxis to all returning pilgrims was not recommended (*7*). However, these findings may not be applicable to pilgrims from developing countries. During the Hajj, pilgrims from poorer countries often live in more overcrowded accommodations than those from more affluent regions, which may increase the risk of droplet transmission and result in higher rates of asymptomatic carriage.

Because several countries do not have scheduled flights to Saudi Arabia, many pilgrims travel on chartered airplanes, which would facilitate the administration of prophylaxis at the airport to returning pilgrims. The effectiveness of single-dose oral ciprofloxacin (*8*) simplifies the task. The disadvantages of ciprofloxacin prophylaxis must, however, be considered, including the small risk for anaphylaxis-like reaction (*9*). The growing problem of antibiotic-resistant organisms cannot be ignored, as fluoroquinolone resistance in the related species *N. gonorrhoeae* has already emerged in many places (*10*). Children and pregnant women, who likely represent only a small proportion of pilgrims, could be given intramuscular ceftriaxone, but there is also a risk of anaphylaxis besides the disadvantages of parenteral therapy. The decision to administer chemoprophylaxis to all returning pilgrims should therefore depend on whether transmission of W135 meningococcus during future pilgrimages continues. Information regarding continuing transmission would be useful before pilgrims return to their homes.

During Hajj 2001, many pilgrims, especially from developing countries, were unlikely to have received the quadrivalent vaccine. The Ministry of Health of Saudi Arabia has recently specified that, beginning in 2002, pilgrims must have been vaccinated with a quadrivalent vaccine (*2*). This requirement may reduce future transmission. However, if cases continue to occur, many countries should consider prophylaxis for returning pilgrims. Clearly, surveillance for meningococcal disease in general and serogroup W135 in particular will remain important in the next few years.
